# Supplementary material for: Unstable anthocyanin pigmentation in Streptocarpus sect. Saintpaulia (African violet) is due to transcriptional selectivity of a single MYB gene
Source: New Phytol. 2025 Jun 13;247(3):1426–44. doi: 10.1111/nph.70286 (PMC12222921; doi:10.1111/nph.70286)
Supplement: Supplementary file 2 — Fig. S1 Cultivars and mutants used in this study. Fig. S2 Developmental stages of petals used in this study. Fig. S3 HPLC analysis of KP and KW petals. Fig. S4 Expression levels of ABGs in white‐striped petals. Fig. S5 Methylation levels of anthocyanin biosynthesis‐related genes in KP and KW leaves. Fig. S6 Predicted promoter sequences of SiMYB2‐Short. Fig. S7 qRT‐PCR analysis of the agroinfiltrated genes in tobacco leaves. Fig. S8 Testing of SiMYB2‐Short repression activity as R3‐MYB. Fig. S9 Transcript variants expression and genome structure of the SiMYB2 gene in several cultivars. [file NPH-247-1426-s002.docx]

## *New Phytologist* Supporting Information

Article title: Unstable anthocyanin pigmentation in *Streptocarpus* sect. *Saintpaulia* (African violet) is due to transcriptional selectivity of a single MYB gene

Authors: Daichi Kurata^1^･Tomohisa Tsuzaki^1^･Fumi Tatsuzawa^2^･Kenta Shirasawa^3^･Hideki Hirakawa^3^･Munetaka Hosokawa^1,4,*^

Article acceptance date: 06 May 2025

The following Supporting Information is available for this article:

**Fig. S1 Cultivars and mutans used in this study.**

**Fig. S2 Developmental stages of petals used in this study.**

**Fig. S3 HPLC analysis of KP and KW petals.**

**Fig. S4 Expression levels of ABGs in white-striped petals.**

**Fig. S5 Methylation levels of anthocyanin biosynthesis related genes in KP and KW leaves.**

**Fig. S6 Predicted promoter sequences of *SiMYB2-Short*.**

**Fig. S7 qRT-PCR analysis of the agroinfiltrated genes in tobacco leaves.**

**Fig. S8 Testing of *SiMYB2-Short* repression activity as R3-MYB.**

**Fig. S9 Transcript variants expression and genome structure of the *SiMYB2* gene in several cultivars.**

**Table S1 List of primers using in this study.**

**Table S2 List of GenPept accession IDs using in phylogenetic tree.**

**Table S3 Result of DMR analysis in Sio_r2.0_p0007.1 contig.**

**Table S4 Location and length of other TE-like sequences that were hit as a result of BLAST using the TIR sequence of MYB2 TE-like as query.**

**Datasets S1 *SiMYB2-Long* sequences.**

**
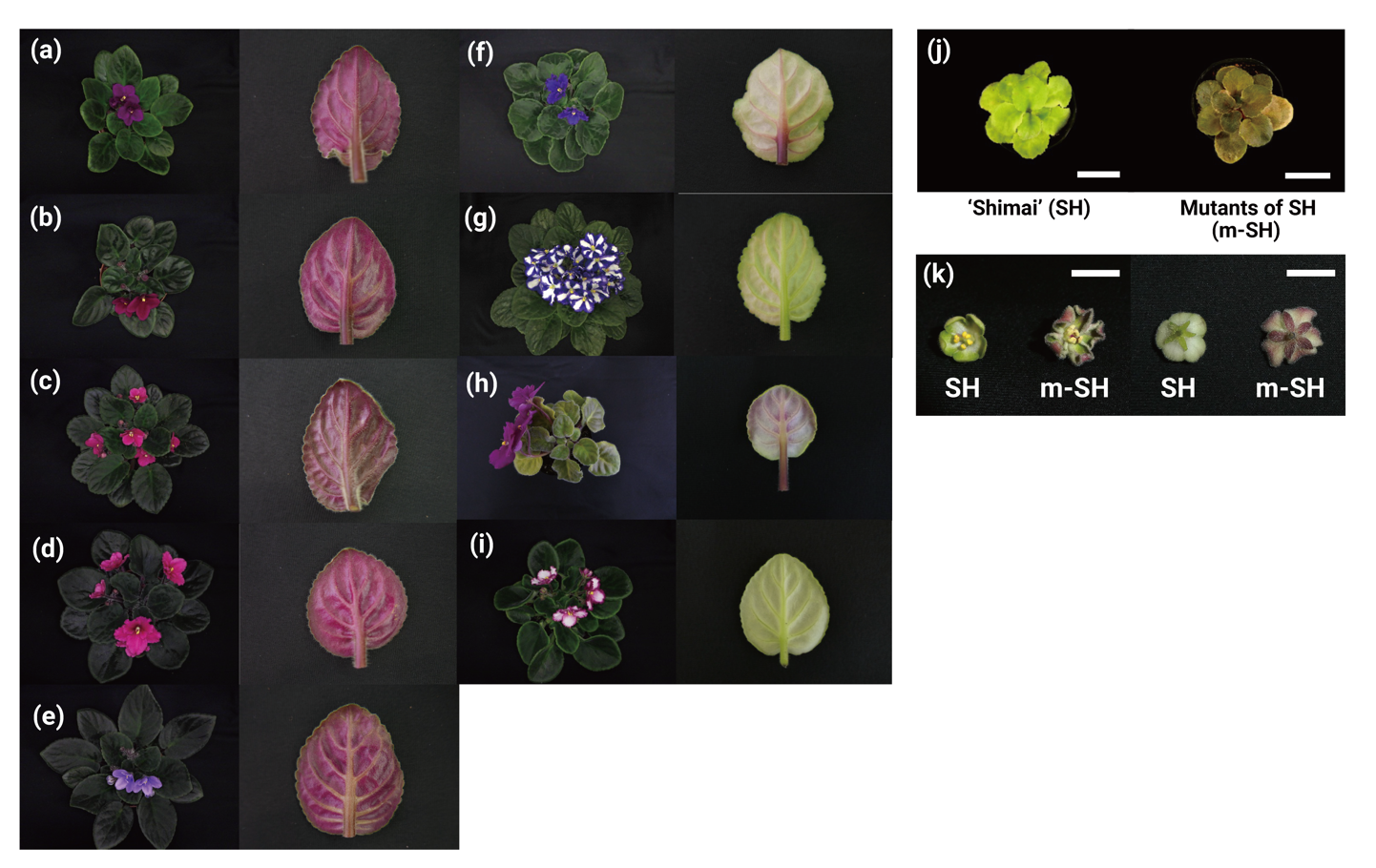
Fig. S1 Cultivars and mutans used in this study.**

(a­–i) Rosette and leaf photos of several cultivars used in this study. (a) ‘Akira’, (b) ‘Tomoko’, (c) ‘Georgia’, (d) ‘Barbara’, (e) ‘Manitoba’, (f) ‘Taro’, (g) ‘Taro’ with white-striped petals, (h) ‘Saturn’ with red petals, (i) ‘Saturn’. (j–k) Rosette and flowers of ‘Shimai’(SH) and mutants of ‘Shimai’(m-SH). (j) White bars indicate 5 cm scale. (k) White bars indicate 1.5 cm scale.

**Fig. S2 Developmental stages of petals used in this study.**

Black bar indicates 1 cm scale. Red bars indicate the stage of petals used in this study.

**Fig. S3 HPLC analysis of KP and KW petals.**

(a,b,e,f) Petal. (c,d,g,h) Leaf. (a–d) 530 nm for anthocyanin. (e–h) 350 nm for flavone. A1: Pelargonidin-3-acetyl-rutinoside-5-glucoside, A2: Peonidin-3-acetyl-rutinoside-5-glucoside, A3: Cyanidin-3-acetyl-sambubioside, F1: Apigenin-4’-glucuronide, F2: Luteolin-4’-glucuronide.

**Fig. S4 Expression levels of ABGs in white-striped petals.**

Asterisks indicate significant differences according to Student’s *t*-test (**P* < 0.05, ***P* < 0.01, ****P* < 0.001). n.s. indicates no significant difference. Error bars represent means ± SD (n = 3). (a) Expression levels in KWS petals. Pink indicates pink regions in KWS petals. White indicates white regions. (b) Expression levels in SWS petals. Purple indicates purple regions in SWS petals. White indicates white regions.

**Fig. S5 Methylation levels of anthocyanin biosynthesis related genes in KP and KW leaves.**

**Fig. S6 Predicted promoter sequences of *SiMYB2-Short*.**

Two types of TATA-boxes reported by Mukumoto et al. (1993) are highlighted. Start codon is indicated in red letters. Predicted 5’ UTR sequences is indicated in blue letters.

**Fig. S7 qRT-PCR analysis of the agroinfiltrated genes in tobacco leaves.**

1: *GFP*, 2: *SiMYB1*, 3: *SiMYB2-Long*, 4: *SiMYB2-Short*, 5: *SibHLH1*, 6: *SiMYB1* + *SibHLH1*, 7: *SiMYB2-Long* + *SibHLH1*, 8: *SiMYB2-Short* + *SibHLH1*. Error bars represent means ± SD (n = 3).

**Fig. S8 Testing of *SiMYB2-Short* repression activity as R3-MYB.**

The ratio indicates *SiMYB1*: *SibHLH1*: *GFP* (left) or *SiMYB1* (right). Tobacco leaves were photographed four days post infiltration. Infiltration was performed using two leaves from each of the three tobacco plants.

**
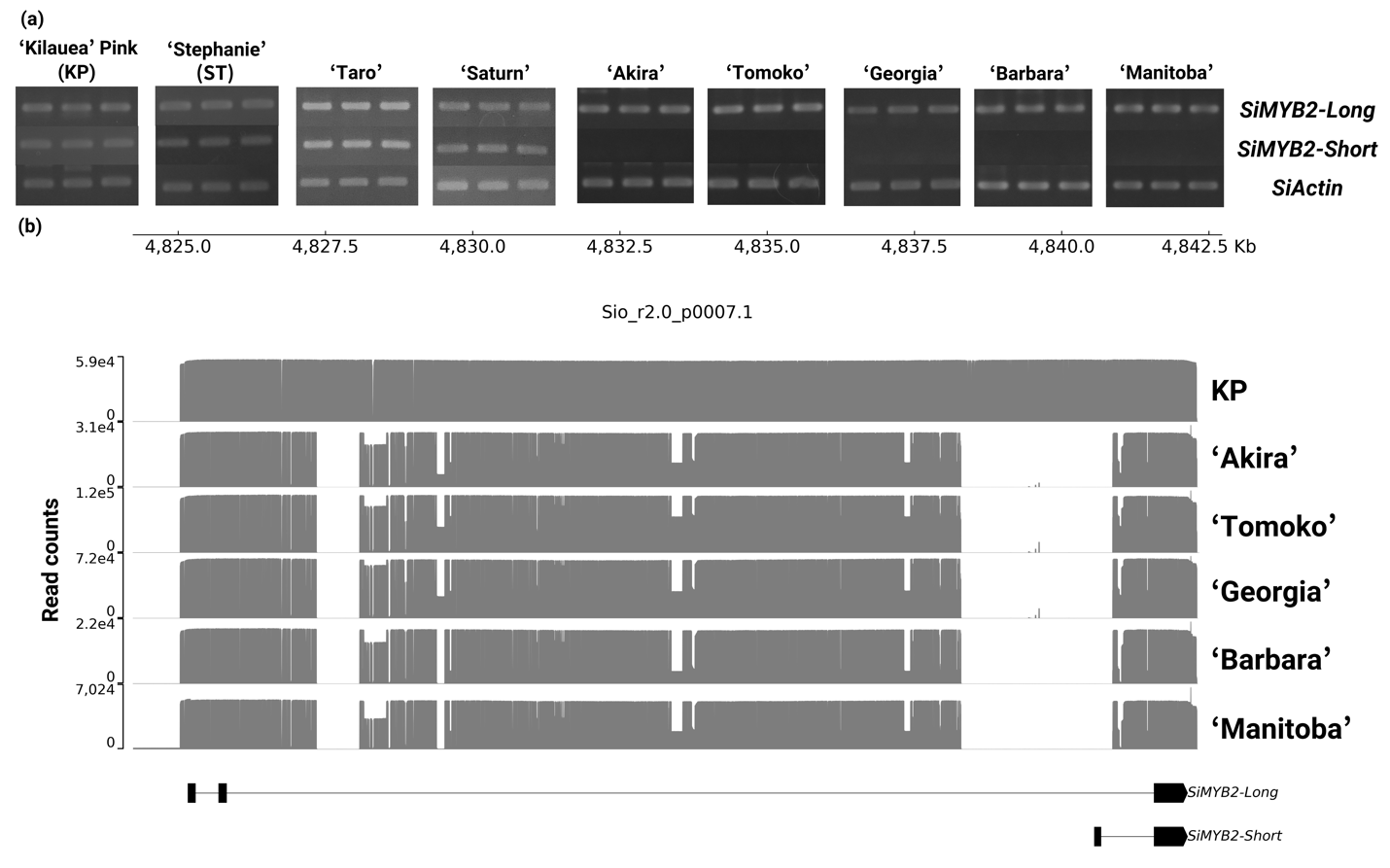
Fig. S9 Transcript variants expression and genome structure of the *SiMYB2* gene in several cultivars.**

(a) RT-PCR results of *SiMYB2* variants in several cultivars. The amplification reaction was carried out for 40 cycles (plateau). Actin was used as control. (b) Amplicon-seq results in several cultivars. The vertical axis indicates read counts and the horizontal axis indicates the position in the Sio_r2.0_p0007.1 contig.
